# CD84 is a Suppressor of T and B Cell Activation during Mycobacterium tuberculosis Pathogenesis

**DOI:** 10.1128/spectrum.01557-21

**Published:** 2022-02-23

**Authors:** Nan Zheng, Joy Fleming, Peilei Hu, Jianjian Jiao, Guoqin Zhang, Ruifang Yang, Chuanyou Li, Yi Liu, Lijun Bi, Hongtai Zhang

**Affiliations:** a Key Laboratory of RNA Biology, Institute of Biophysics, Chinese Academy of Sciences, Beijing, China; b Hunan Chest Hospital, Changsha, Hunan Province, China; c Beijing Chest Hospital, Capital Medical University, Beijing Tuberculosis and Thoracic Tumor Research Institute, Beijing Key Laboratory for Drug Resistant Tuberculosis Research, Beijing, China; d CAS Center of Excellence in Biomacromolecules, Institute of Biophysics, Chinese Academy of Sciences, Beijing, China; e Guangdong Province Key Laboratory of TB Systems Biology and Translational Medicine, Foshan, Guangdong Province, China; f University of Chinese Academy of Sciences, Beijing, China; National Institutes of Health

**Keywords:** CD84, checkpoint immunotherapy, *Mycobacterium tuberculosis*, pathogenesis

## Abstract

Interest in host-directed therapies as alternatives/adjuncts to antibiotic treatment has resurged with the increasing prevalence of antibiotic-resistant tuberculosis (TB). Immunotherapies that reinvigorate immune responses by targeting immune checkpoints like PD-1/PD-L1 have proved successful in cancer therapy. Immune cell inhibitory receptors that trigger Mycobacterium tuberculosis-specific immunosuppression, however, are unknown. Here, we show that the levels of CD84, a SLAM family receptor, increase in T and B cells in lung tissues from M. tuberculosis-infected C57BL/6 mice and in peripheral blood mononuclear cells (PBMCs) from pulmonary TB patients. M. tuberculosis challenge experiments using CD84-deficient C57BL/6 mice suggest that CD84 expression likely leads to T and B cell immunosuppression during M. tuberculosis pathogenesis and also plays an inhibitory role in B cell activation. Importantly, CD84-deficient mice showed improved M. tuberculosis clearance and longer survival than M. tuberculosis-infected wild-type (WT) mice. That CD84 is a putative M. tuberculosis infection-specific inhibitory receptor suggests it may be a suitable target for the development of TB-specific checkpoint immunotherapies.

**IMPORTANCE** Immune checkpoint therapies, such as targeting checkpoints like PD-1/PD-L1, have proved successful in cancer therapy and can reinvigorate immune responses. The potential of this approach for treating chronic infectious diseases like TB has been recognized, but a lack of suitable immunotherapeutic targets, i.e., immune cell inhibitory receptors that trigger immunosuppression specifically during Mycobacterium tuberculosis pathogenesis, has limited the application of this strategy in the development of new TB therapies. Our focus in this study was to address this gap and search for an M. tuberculosis-specific checkpoint target. Our results suggest that CD84 is a putative inhibitory receptor that may be a suitable target for the development of TB-specific checkpoint immunotherapies.

## INTRODUCTION

Tuberculosis (TB), caused by the pathogen Mycobacterium tuberculosis, is one of the top 10 causes of death worldwide and the leading cause of death from a single infectious agent (1). With an estimated 10 million incident cases globally each year and significant increases in TB cases and deaths expected in coming years due to the heavy impact of the COVID-19 pandemic on TB programs (2), TB is a major burden on public health systems. The increasing prevalence of antibiotic-resistant TB (an estimated 500,000 cases worldwide in 2019, ∼78% of which were multidrug resistant [MDR]) (3) and associated reductions in treatment success rates (drug-sensitive [DS] TB, 85%; MDR TB, 56%; extensively drug-resistant [XDR] TB, 39%) (3) have led to a resurgence of interest in a range of alternative host-directed therapeutic strategies that modulate host cell responses to support pathogen clearance, thus improving clinical outcomes for TB patients (4). Immune checkpoint blockade has been shown to reinvigorate immune responses (5), and following its successful application in cancer therapies, such as the targeting of the PD-1/PDL-1 checkpoint in lung cancer immunotherapies (6), is considered a potential therapeutic approach for boosting T cell immunity in chronic infections like TB (7). Although the roles of immune checkpoint molecules like PD-1, CTLA-4, and TIM-3 in M. tuberculosis pathogenesis have been investigated (8–11), to date, suitable TB-specific immune checkpoint targets have yet to be identified.

The ability of M. tuberculosis to infect host cells and maintain long-term persistent infections is due in large part to its ability to evade host immune responses (12). An effective host immune response involving both cell-mediated and humoral responses is thus crucial for providing protection against M. tuberculosis infection and subsequent disease development. Innate responses, such as the production of reactive oxygen species (ROS) and reactive nitrogen species (RNS) and killing of intracellular pathogens via phagosomes or autophagy, are also important. The CD4^+^ T cell response plays a critical role in protective immunity against M. tuberculosis infection and is characterized by Th1 cells that secrete interferon gamma (IFN-γ) and other cytokines to activate macrophages that have phagocytosed the pathogen and promote the formation of granulomas (13, 14). Immune cells like CD8^+^ T cells, γδ T cells, and CD-1-restricted T cells also have important roles (15). B cells and antibodies also significantly affect the development of immune responses to M. tuberculosis and can modulate local control of infection (16, 17). Dysfunction in the host immune response negatively affects its ability to kill and clear intracellular M. tuberculosis, and immunotherapies that modulate the immune responses of those latently infected with M. tuberculosis or with active TB disease may enable better control of bacterial replication (4).

T cell immune responses are regulated by different stimulatory and inhibitory surface receptor proteins, referred to as immune checkpoint proteins (18). Costimulatory and coinhibitory proteins are involved in the fine-tuning of immune signals; costimulators tune up an immune signal, while coinhibitors tune it down (19). Immune checkpoint proteins used in therapeutic approaches are inhibitory receptors that promote inhibitory interactions between immune cells and trigger immunosuppressive signaling pathways (20). Effector T cells and other immune cells can be driven into a state of exhaustion by sustained signaling via immune checkpoint proteins, reducing their effector function and ultimately leading to immune control escape (21). Therapeutic blockade of immune checkpoint protein PD1, CTAL-4, or TIM-3 has been shown to reverse T cell exhaustion in various cancers, improve antitumor T cell responses, diminish tumor size, and increase survival (22, 23). With respect to the application of immune checkpoint blockade therapies for improving outcomes for TB patients, however, the results of studies to date based on murine M. tuberculosis challenge models have been conflicting. For example, M. tuberculosis-infected PD-1-deficient mice show more severe disease pathology, and the differentiation of PD-1^−^ CD4^+^ T cells is sufficient to trigger early mortality (24, 25). PD1-deficient mice show uncontrolled lung bacterial proliferation and the presence of focal necrotic areas with predominantly neutrophilic infiltrates (24). While TIM-3 blockade is reported to restore T cell functions and improve control of bacterial load in chronically infected susceptible mice (9), TIM-3-expressing T cells from patients with pulmonary TB produce the cytokines IFN-γ/tumor necrosis factor alpha (TNF-α)/interleukin-22 (IL-22), and function as effector cells limiting intracellular M. tuberculosis growth in macrophages (26). A major challenge in the development of checkpoint-based immunotherapies for TB is to identify effective M. tuberculosis-specific checkpoint inhibitory receptors that could ameliorate TB pathogenesis when blockaded.

Here, we set out to identify candidate immune checkpoint target proteins suitable for developing TB-specific immune checkpoint blockade immunotherapies. Transcriptome analysis, quantitative PCR (qPCR), and flow cytometry all indicated that CD84 expression on immune cells was elevated in M. tuberculosis H37Rv-infected mice and in peripheral blood mononuclear cells (PBMCs) from pulmonary TB patients relative to their levels in controls. Further analysis using CD84-deficient mice suggests that CD84 acts as an inhibitory receptor during M. tuberculosis pathogenesis, inhibiting both T and B cell immune responses, limiting M. tuberculosis bacterial clearance, and shortening survival. Similarly, CD84^−^ T cells from pulmonary TB patients also produced significantly more of the anti-M. tuberculosis cytokine IFN-γ. Our data suggest that CD84 is a potential immune checkpoint target protein that could be used in the design of M. tuberculosis-specific immune checkpoint blockade therapies.

## RESULTS

### Expression of signaling lymphocyte activation molecule (SLAM) family genes increases significantly during M. tuberculosis pathogenesis.

**FIG 1:**
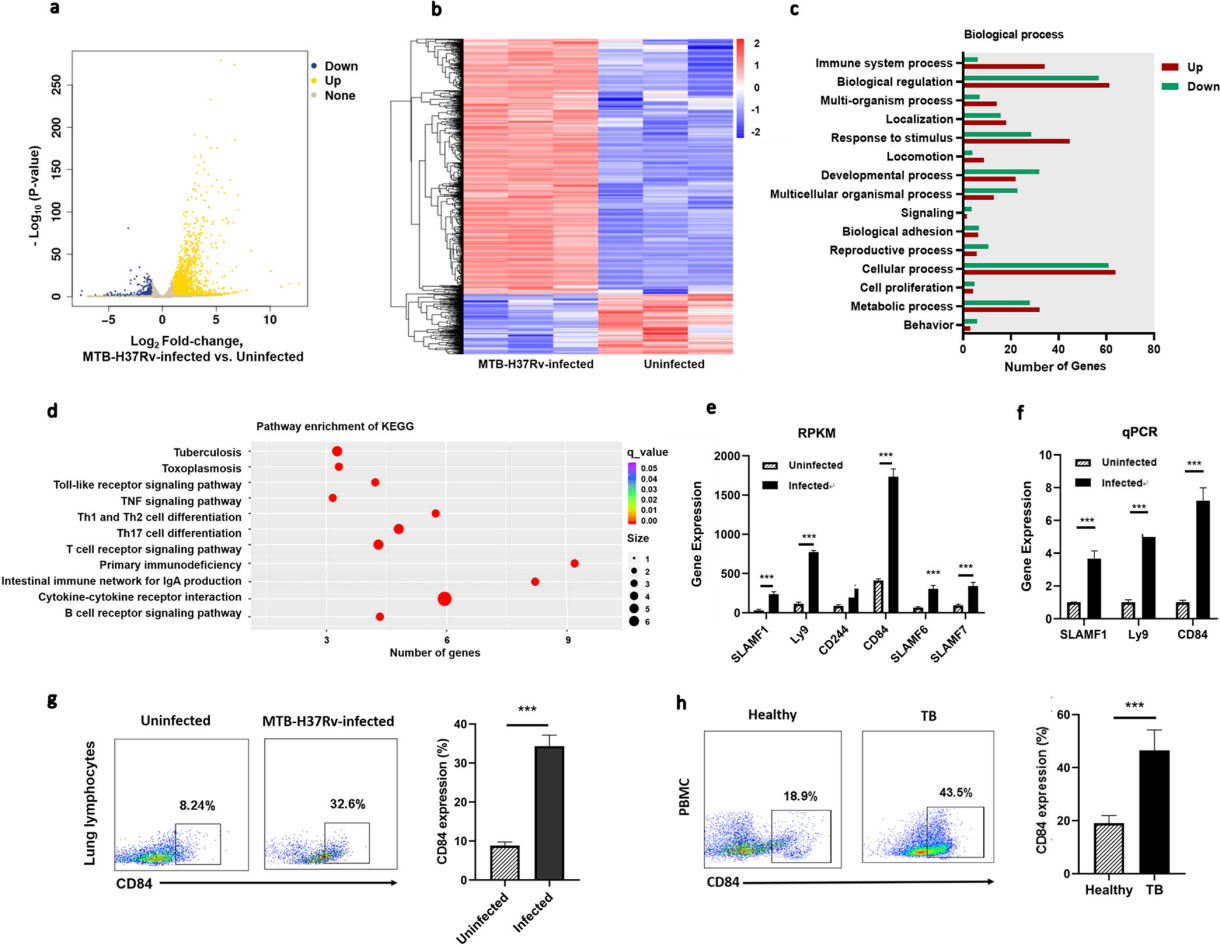
SLAM family member CD84 is more highly expressed in the lungs of M. tuberculosis H37Rv-infected C57BL/6 mice than in the lungs of uninfected mice and in PBMCs from pulmonary TB patients than in PBMCs from healthy donors. (a to e) Total RNA was extracted from lung tissue from M. tuberculosis H37Rv-infected (1 × 10^6^ CFU) and uninfected C57BL/6 mice (*n* = 3) 30 days postinfection and was sequenced on an Illumina HiSeq X Ten system (three independent biological replicates). (a) Scatterplot of the expression of all genes. (b) Heat map of genes specifically upregulated (1,499) and downregulated (361) in the lung transcriptome of mice infected with M. tuberculosis. (c) GO classifications of differentially expressed genes. (d) KEGG enrichment of differentially expressed genes. (e) RNA-Seq data (reads per kilobase per million [RPKM] values) for the expression of SLAMF family proteins in lung tissue from M. tuberculosis-infected and uninfected mice. (f) qPCR analysis of SLAMF1, Ly9, and CD84 expression in lung tissue from M. tuberculosis-infected and uninfected mice, 30 days postinfection. (g) Flow cytometry of lung lymphocytes from M. tuberculosis-infected and uninfected mice stained by PE–anti-CD84 antibody; histogram showing the percentages of lung lymphocytes expressing CD84. (h) Flow cytometry of PBMCs derived from patients with pulmonary TB and healthy donors and stained with PE–anti-CD84 antibody; histogram showing the percentages of PBMCs expressing CD84. Data presented are mean values ± SD from three independent experiments. ***, *P* < 0.001 (Student’s *t* test).

The development of suitable immune checkpoint blockade immunotherapies for TB requires the identification of immune checkpoint target proteins that are inhibitory receptors whose expression on immune cells increases specifically during M. tuberculosis pathogenesis, promoting immunosuppressive signaling pathways (27, 28). To identify such cosignaling molecules for further analysis, we first obtained a profile of gene transcription associated with pulmonary TB in mice, performing transcriptome sequencing (RNA-Seq) on total RNA from the lung tissue of uninfected control and M. tuberculosis strain H37Rv-infected (1 × 10^6^ CFU) C57BL/6 mice 30 days postinfection. A total of 1,499 genes were upregulated and 361 were downregulated (fold change of ≥2, *P* < 0.05) in infected lung tissue relative to their expression in the uninfected control (Fig. 1a and b). Gene ontology (GO) analysis showed that differentially expressed genes were significantly enriched in immune system processes (Fig. 1c), and KEGG analysis pointed to enrichment of T cell receptor signaling pathway and T cell-related differentiation pathway genes (Fig. 1d).

Cosignaling molecules associated with the initiation and direction of T cell immunity were differentially expressed in lung tissue from M. tuberculosis H37Rv-infected mice (Table S1 in the supplemental material); in particular, SLAM family genes, known to regulate T cell activation (29), were significantly upregulated (Fig. 1e), and qPCR validation of the expression of SLAMF1, Ly9, and CD84 confirmed their increased expression (Fig. 1f), increases in CD84 gene transcripts being the most prominent. Flow cytometry of lung lymphocytes showed that H37Rv-infected mice had a higher proportion of CD84^+^ lung lymphocytes than uninfected mice at 30 days postinfection (Fig. 1g). To determine if these findings had any bearing on the pathogenesis of TB disease in humans, we compared PBMCs derived from pulmonary TB patients and healthy donors and found that the CD84 receptor was expressed at a higher level in PBMCs from TB patients than in those from healthy donors (Fig. 1h). These results suggest that the CD84 receptor may be involved in the immune response to M. tuberculosis in both mice and humans.

### CD84 is more highly expressed in T cells from M. tuberculosis-infected mice than in T cells from uninfected mice and in T cells from pulmonary TB patients than in T cells from healthy donors.

**FIG 2:**
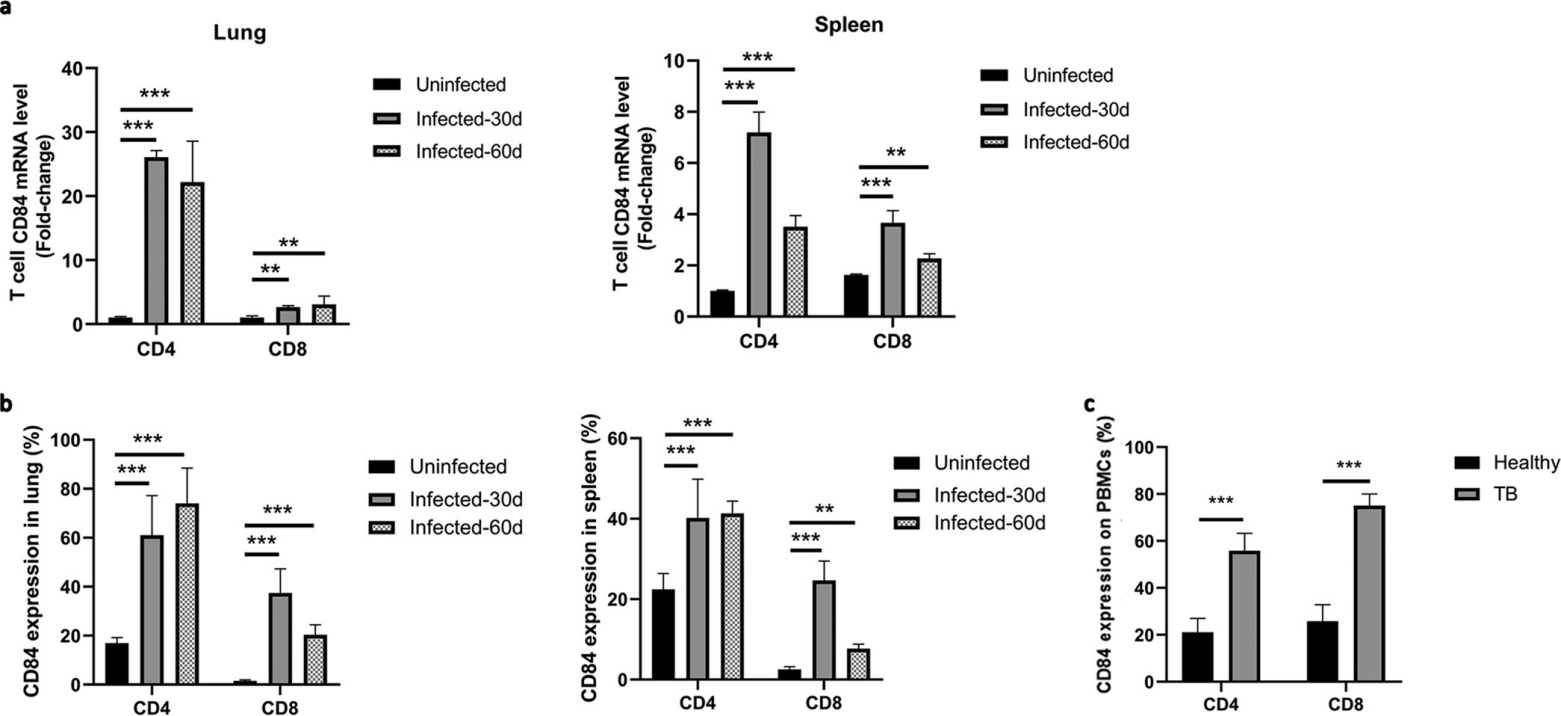
CD84 is more highly expressed on T cells from M. tuberculosis-infected than on T cells from uninfected mice and on PBMCs from pulmonary TB patients than on PBMCs from healthy donors. (a, b) Total RNA was extracted from T cells separated from the lung and spleen tissues of M. tuberculosis-infected (1 × 10^6^ CFU H37Rv) and uninfected C57BL/6 mice 30 days and 60 days postinfection. (a) Levels of CD84 mRNA in CD4^+^ and CD8^+^ T cells from the lung and spleen of M. tuberculosis-infected and uninfected mice, as determined by qPCR. (b) Histogram showing the percentages of CD4^+^ and CD8^+^ T cells expressing CD84 in lung and spleen tissues from M. tuberculosis-infected and uninfected mice, as determined by flow cytometry. (c) Flow cytometry of PBMCs from patients with pulmonary TB and healthy donors, stained by PerCP–anti-CD4 antibody, APC–anti-CD8 antibody, and PE–anti-CD84 antibody. Data presented are mean values ± SD from three independent experiments; *n* = 3 for each group in each experiment. **, *P < *0.01; ***, *P* < 0.001 (Student’s *t* test).

T cells actively contribute to the maintenance of host defenses against M. tuberculosis infection (30). CD84 and other members of the SLAM family of T cell receptors are involved in the activation and regulation of T cells and NK cells (31). When we compared the expression of CD84 on T cells in lung and spleen tissue taken from mice in the above-described experiment after infection with M. tuberculosis for 30 or 60 days to its expression in uninfected control mice, we found that CD84 mRNA levels (determined by qPCR) were significantly higher in CD4^+^ and CD8^+^ T cells in both tissues after M. tuberculosis infection (Fig. 2a). Flow cytometry confirmed that CD4^+^ and CD8^+^ T cells in both lung and spleen tissues displayed increased expression of CD84 (Fig. 2b). Similarly, PBMCs derived from patients with pulmonary TB displayed higher expression of CD84 than those from healthy donors (Fig. 2c). These findings suggest that CD84 may be a receptor involved in generating T cell immune responses during M. tuberculosis pathogenesis.

### CD84 deficiency activates T cell immune responses during M. tuberculosis pathogenesis.

**FIG 3:**
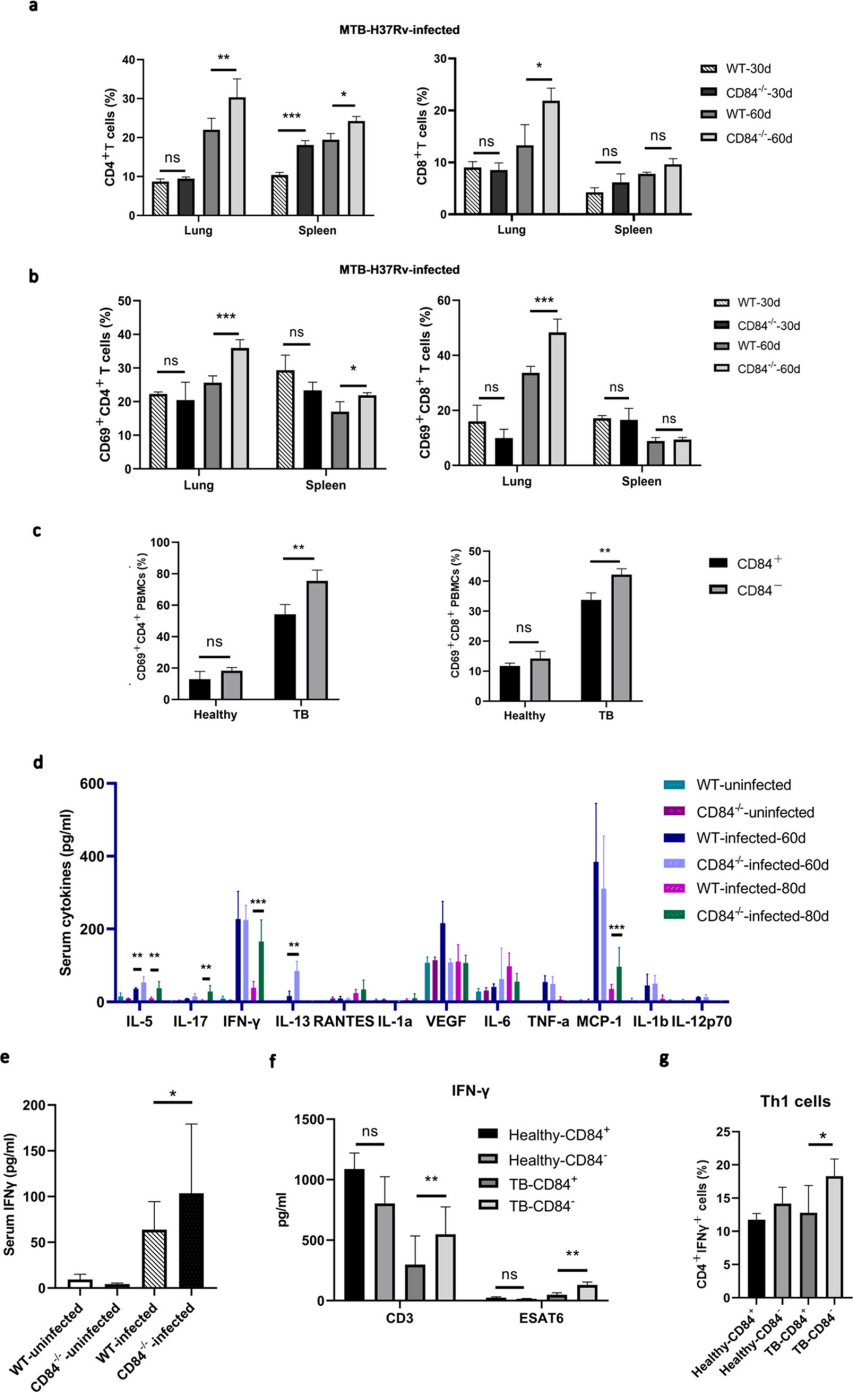
CD84 deficiency activates T cell immune responses during M. tuberculosis pathogenesis. (a, b) T cells were isolated from lung and spleen tissues from M. tuberculosis-infected (1 × 10^6^ CFU strain H37Rv) and uninfected WT and CD84-deficient C57BL/6 mice 30 and 60 days postinfection. (a) Percentages of CD4^+^ and CD8^+^ T cells in lung and spleen tissues from M. tuberculosis-infected WT or CD84-deficient mice 60 days postinfection, as determined by flow cytometry. (b) Percentages of CD69^+^ CD4^+^ and CD69^+^ CD8^+^ T cells in lung and spleen tissues from M. tuberculosis-infected WT and CD84-deficient mice 60 days postinfection, as determined by flow cytometry. (c) Percentages of CD84^+^ or CD84^−^ CD4^+^ and CD8^+^ T cells from pulmonary TB patients and healthy donors, as determined by flow cytometry after staining with PerCP–anti-CD4 antibody, APC–anti-CD8 antibody, and FITC–anti-CD69 antibody. (d) Cytokine levels in serum from M. tuberculosis-infected and uninfected WT and CD84-deficient mice 60 days and 80 days postinfection. Quantibody multiplexed quantitative sandwich ELISA array data for 12 TB-related cytokines. (e) Validation of IFN-γ expression at 80 days postinfection using standard ELISAs. (f) IFN-γ release from CD84^+^ and CD84^−^ PBMCs from pulmonary TB patients and healthy donors as determined using ELISA. CD84^+^ and CD84^−^ T cells were activated with soluble anti-CD3 antibody (clone OKT3) and ESAT-6 (5 μg/mL). (g) IFN-γ expression in differentiated Th1 cells as determined by flow cytometry. Sorted naive CD4^+^ T cells derived from PBMCs were cultured for 72 h under Th1-differentiating conditions and then stimulated for 5 h with 2 μL/mL cell activation cocktail. Data correspond to mean values ± SD from three independent experiments; *n* = 3 for each group in each experiment. *, *P < *0.05; **, *P < *0.01; ***, *P* < 0.001; ns, nonsignificant (Student’s *t* test).

CD84 can act as an activating or inhibitory receptor in immune cell activation depending on the cell type and stage of activation (32, 33), but its involvement in M. tuberculosis pathogenesis has not previously been reported. As the T cell exhaustion observed in chronic TB infections is characterized by elevated expression of multiple inhibitory receptors like PD-1, TIM-3, LAG-3, and CTLA-4, progressive failure of T cell activation, and decreased production of cytokines like IFN-γ (9, 34), we sought to determine if the role CD84 plays in T cell activation during M. tuberculosis pathogenesis is inhibitory and may thus contribute to T cell exhaustion. We created a CD84-deficient mouse model (C57BL/6N) by CRISPR/Cas-mediated genome engineering (Fig. S1), and in agreement with previous reports, found that these CD84-deficient mice showed normal development of conventional CD4^+^ and CD8^+^ T cells (Fig. S2) (31, 35), indicating that CD84 deficiency does not affect T cell development under normal conditions. We then analyzed the role of CD84 during M. tuberculosis H37Rv infection, first by measuring its expression on T cell subsets in lung and spleen tissues from mice infected with M. tuberculosis H37Rv over a period of 60 days. Significantly higher numbers of CD4^+^ cells were present in the lung and spleen tissues of CD84-deficient infected mice at day 60 postinfection than in wild-type (WT) mice (Fig. 3a), and CD8^+^ T cells were significantly elevated in the lung. We then investigated whether the expression on CD4^+^ and CD8^+^ T cells of CD69, an early activation marker on T cells that is considered to be a reliable measure of lymphocyte activation (36), was increased in CD84-deficient mice during M. tuberculosis pathogenesis. Flow cytometry showed that CD69 was expressed at significantly higher levels on CD4^+^ and CD8^+^ T cells from these CD84-deficient infected mice at 60 days postinfection than in the WT infected controls (Fig. 3b). In contrast, in agreement with other reports (35), in the absence of M. tuberculosis infection, CD4, CD8, and CD69 expression did not differ between CD84-deficient and WT mice (Fig. S2). A similar phenomenon was observed in human PBMCs from pulmonary TB patients and healthy donors; when CD84^−^ CD4^+^, CD84^−^ CD8^+^, CD84^+^ CD4^+^, and CD84^+^ CD8^+^ T cells were sorted and analyzed by flow cytometry, we found significantly higher expression of CD69 on CD84^−^ CD4^+^ and CD84^−^ CD8^+^ T cells than on CD84^+^ CD4^+^ and CD84^+^ CD8^+^ T cells (Fig. 3c).

Activated T cells respond to infection by releasing multiple cytokines and chemokines during M. tuberculosis pathogenesis (37). When we assessed cytokine levels in sera from infected mice at 60 days and 80 days postinfection using Quantibody multiplexed quantitative sandwich enzyme-linked immunosorbent assay (ELISA) arrays (38), we detected higher levels of IL-5, IL-17, IFN-γ, and MCP-1 in infected CD84-deficient mice than in infected WT mice at 80 days postinfection (Fig. 3d). Interestingly, while there was no difference in IFN-γ levels between WT and CD84-deficient mice at 60 days postinfection (the levels in both cases being substantially raised relative to the levels in uninfected mice), there was a marked drop in the IFN-γ level by 80 days postinfection in WT mice (suggestive of the onset of T cell exhaustion), while the IFN-γ in CD84-deficient mice was maintained at a similar level. Validation of IFN-γ levels using a standard ELISA (Fig. 3e) confirmed the trends observed. Similarly, the levels of IFN-γ released from CD84^−^ PBMCs from pulmonary TB patients after anti-CD3 or ESAT-6 stimulation were also significantly higher than the levels released from CD84^+^ PBMCs (Fig. 3f), indicating that CD84 can suppress the IFN-γ release in TB patients that is associated with a Th1 immune response against M. tuberculosis infection (39). Flow cytometry of IFN-γ expression after culturing naive CD4^+^ T cells under Th1 differentiation conditions showed that the percentage of CD4^+^ IFN-γ^+^ Th1 cells derived from naive CD84^−^ CD4^+^ T cells was higher than that from naive CD84^+^ CD4^+^ T cells (Fig. 3g), suggesting that CD84 plays an inhibitory role in the differentiation and regulation of Th1 cells.

Taken together, these results demonstrate that CD84 deficiency activates T cell immune responses, and thus, CD84 is likely an inhibitory receptor during M. tuberculosis pathogenesis.

### CD84 deficiency activates the B cell response during M. tuberculosis pathogenesis in mice.

**FIG 4:**
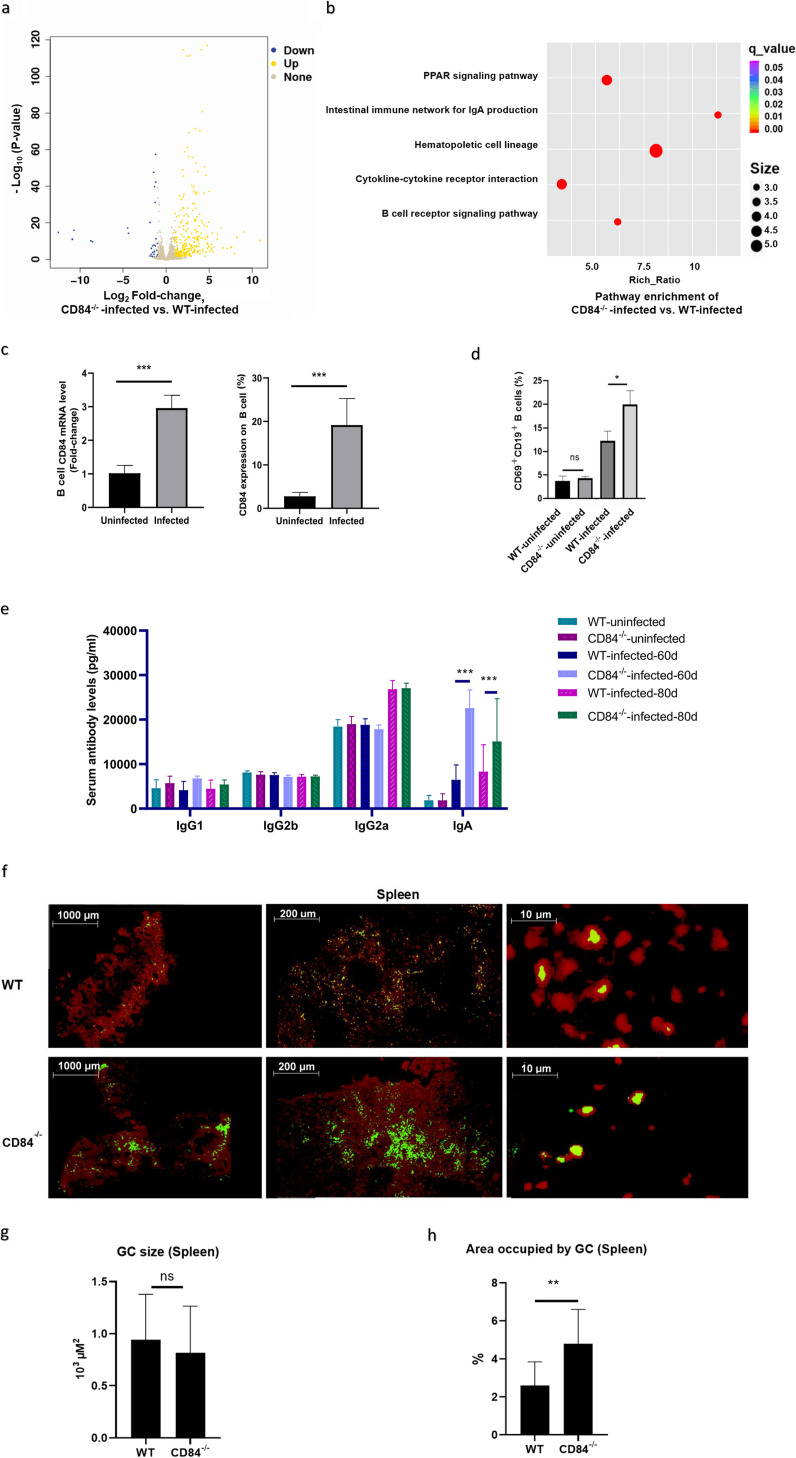
CD84 inhibits B cell activation and antibody production in response to M. tuberculosis infection in mice. (a to c) RNA-Seq of total RNA from lung tissues from M. tuberculosis-infected (1 × 10^6^ CFU strain H37Rv) WT and CD84-deficient C57BL/6 mice 30 days postinfection. (a) Scatterplot of the expression of all genes. (b) KEGG enrichment of differentially expressed genes. (c) qPCR determination of CD84 mRNA levels in B cells (left) and percentages of CD84^+^ B cells in spleen tissues from M. tuberculosis-infected and uninfected WT and CD84-deficient mice 30 days postinfection (right), as determined by flow cytometry. (d) CD69 expression on B cells in spleen tissue from M. tuberculosis-infected and uninfected WT and CD84-deficient mice 60 days postinfection, as determined by flow cytometry. (e) Antibody levels in sera from M. tuberculosis-infected and uninfected WT and CD84-deficient mice 60 days and 80 days postinfection, as determined using Quantibody multiplexed quantitative sandwich ELISA arrays. (f) Immunohistology of spleen tissue from M. tuberculosis-infected (1 × 10^6^ CFU H37Rv) WT and CD84-deficient C57BL/6 mice 60 days postinfection, stained with GC B cell markers PE-IgD (red) and FITC-GL7 (green). (g, h) Average GC size (g) and percentage of area occupied by GCs (h) as quantified using a Zeiss Axioplan microscope morphometric tool. Data presented are mean values ± SD from three independent experiments; *n* = 3 for each group in each experiment. *, *P* < 0.05; **, *P* < 0.01; ***, *P* < 0.001; ns, nonsignificant (Student’s *t* test).

To further understand the function of CD84 during M. tuberculosis pathogenesis, we characterized the transcriptomes of M. tuberculosis H37Rv-infected WT and CD84-deficient mice 30 days postinfection, using RNA-Seq. A total of 448 genes were specifically upregulated and 105 were downregulated (≥2-fold change) in lung tissue from CD84-deficient mice compared to their expression in WT mice (Fig. 4a). KEGG analysis of differentially expressed genes indicated that B cell receptor signaling pathways were among the significantly enriched pathways (Fig. 4b).

B cells modulate the immune response to various intracellular pathogens, including M. tuberculosis. However, experiments with CD84-deficient mice (in the absence of infection) have shown that B cell development is not affected by CD84 deficiency and that CD84-deficient B cells respond normally to antigen or mitogen stimulation *in vitro* and *in vivo* and do not show altered antibody production (35). Here, both qPCR analysis and flow cytometry indicated that CD84 was expressed at higher levels in B cells from spleen tissues in WT mice infected with M. tuberculosis than those in uninfected mice at 60 days postinfection (Fig. 4c). While, similar to the above report (35), the numbers of B cells expressing the early activation marker CD69 were not significantly different between WT and CD84-deficient cells in the absence of M. tuberculosis infection, the CD69 expression was significantly higher in B cells from CD84-deficient mice than in those from WT mice at 60 days post-M. tuberculosis infection (Fig. 4d; Fig. S3), implying that CD84 inhibits the activation of M. tuberculosis infection-associated B cells and, thus, modulates the immune response. Immunoglobulin A (IgA) deficiency is known to increase susceptibility to M. tuberculosis infection in mice (40, 41), likely because IgA is known to be involved in controlling and eradicating pathogen infections through a variety of antibody-mediated innate effector cell mechanisms (42). Here, the serum levels of IgA, but not IgG1, IgG2b, or IgG2a, produced during M. tuberculosis pathogenesis were higher in CD84-deficient mice than in WT mice 60 and 80 days postinfection (Fig. 4e).

Germinal centers (GCs) are specialized lymphoid microenvironments that generate high-affinity antibodies during T cell-dependent B cell responses (43). GCs form in lymph node follicles and are the sites of B cell activation, proliferation, Ig class switching, and somatic hypermutation leading to increased antigen affinity (44, 45). TB is a chronic infection, and the formation of GCs within the granuloma permits more efficient antigen presentation and lymphocyte activation (46). CD84 is known to be involved in the T cell-B cell interactions required for delivery of T cell help and GC formation, and CD84 deficiency has been reported to affect GC morphology (47). When we performed immunohistological staining of lung and spleen tissues to investigate whether CD84 deficiency affects the structure of the GC during M. tuberculosis infection, we noted larger GC clusters in tissues from M. tuberculosis H37Rv-infected CD84-deficient mice than in tissues from infected WT mice at 60 days postinfection (Fig. 4f; Fig. S4). Contrary to the above-mentioned study performed in the absence of infection and suggesting that B cell development and antibody production are not affected by CD84 deficiency (35), morphometric analysis here indicated that, while the average size of GCs was the same in M. tuberculosis-infected CD84-deficient and WT mice at 60 days postinfection, the area occupied by GC clusters was greater in M. tuberculosis-infected CD84-deficient mice than in M. tuberculosis-infected WT mice (Fig. 4g and h). After M. tuberculosis infection, the GCs of CD84-deficient mice appeared to aggregate into clusters and occupy a larger area than in WT mice.

Taken together, these results suggest that CD84 deficiency promotes the activation of B cells and the aggregation of GCs in mice during M. tuberculosis pathogenesis. Increases in GC aggregation may improve the efficacy of the humoral immune response against M. tuberculosis infection as reflected in increased IgA production, facilitating pathogen clearance.

### CD84 deficiency improves M. tuberculosis clearance and survival.

**FIG 5:**
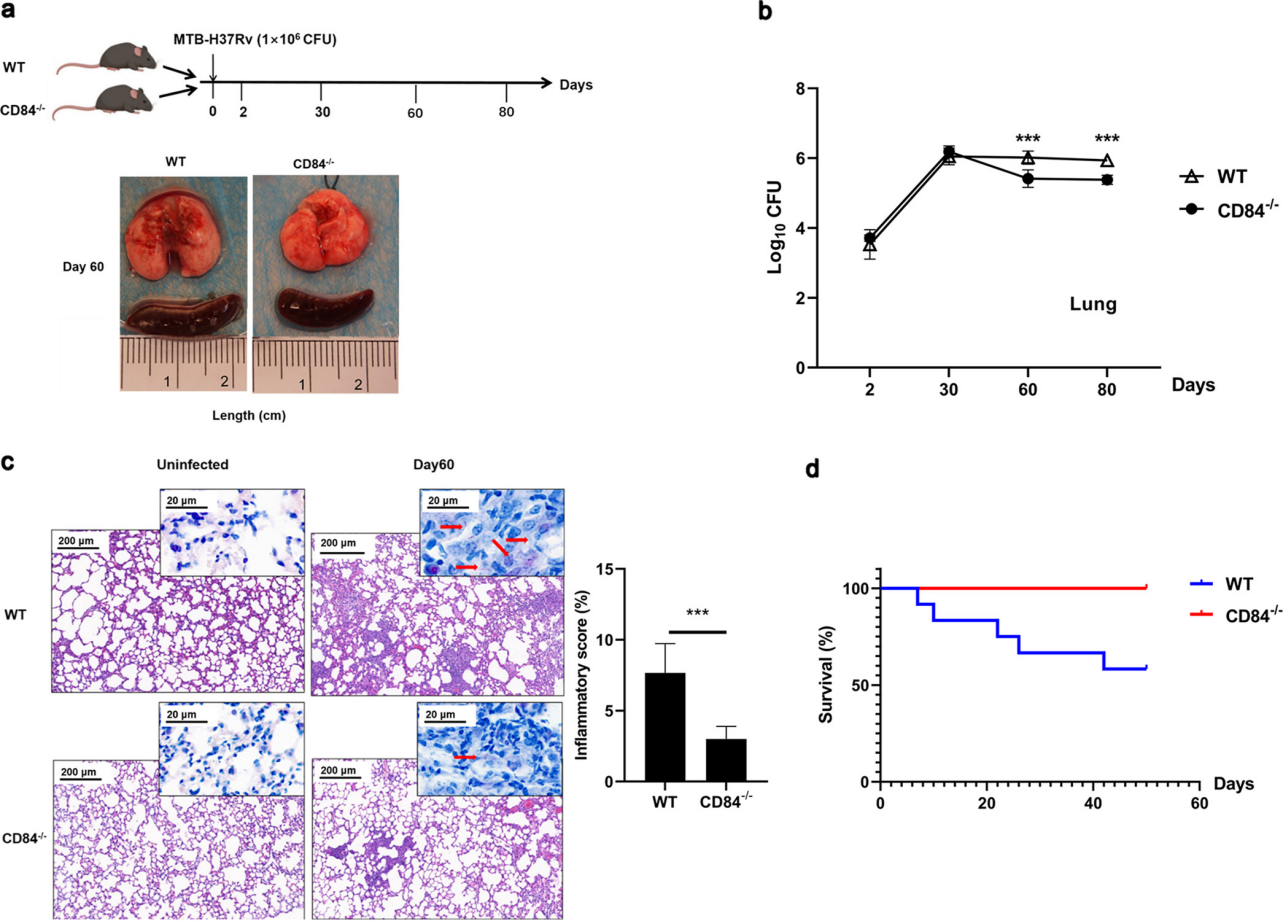
CD84 deficiency may promote M. tuberculosis clearance and survival. (a) Lung and spleen necroscopy of M. tuberculosis-infected (∼1 × 10^6^ CFU strain H37Rv) CD84-deficient and WT C57BL/6 mice 60 days postinfection. (b) Lung bacterial loads for M. tuberculosis-infected CD84-deficient and WT mice at the time points indicated (postinfection). (c) Left, H&E staining (×10 magnification, scale bar = 200 μm) and inset images of AF-stained lung tissue samples (×100 magnification, scale bar = 20 μm) for M. tuberculosis-infected CD84-deficient and WT mice showing lung pathology and the presence of M. tuberculosis bacilli in lung tissues 60 days postinfection. Right, histological score (area occupied by inflammation as a percentage of the total surface area in H&E-stained lung tissue sections). (d) Survival curve for a chronic infection model in which WT and CD84-deficient mice (*n* = 12) were infected with ∼1 × 10^8^ CFU M. tuberculosis H37Rv. Data presented in panels b and c represent mean values ± SD from representative experiments; *n* = 6 mice per group, three independent biological replicates being used in each case. ***, *P* < 0.001; ns, nonsignificant (Student’s *t* test).

To determine if CD84 deficiency-related activation of T and B cells affected M. tuberculosis clearance in the lung, we compared lung pathology and CFU counts in M. tuberculosis H37Rv-infected CD84-deficient and WT mice over a period of 80 days (Fig. 5a and b). The lung CFU counts were significantly lower in lung, but not spleen, tissue from CD84-deficient mice than in WT mice by 60 days postinfection (CD84-deficient mice, 2.42E+05 ± 2.217E+05 CFU [mean ± standard deviation], and WT mice, 1.12E+06 ± 5.046E+05 CFU [*P* < 0.001, two-tailed unpaired *t* test]) (Fig. 5b). Less extensive tissue damage was observed in lung tissues from infected CD84-deficient mice than in those from infected WT mice at 60 days postinfection: tissue sections (hematoxylin and eosin Y [H&E] and acid-fast [AF] staining) showed reduced infiltration of inflammatory cells in peribronchial areas in CD84-deficient mice and less pulmonary hemorrhage, lymphocyte infiltration of blood vessels and bronchi, foam cell aggregation, and inflammatory granuloma formation in the lungs. Associated histological scores (based on the percentage of the total area occupied by inflammation) supported these findings (Fig. 5c). In addition, to investigate whether CD84 affects survival, we infected WT and CD84-deficient mice with a higher dose of M. tuberculosis H37Rv (1 × 10^8^ CFU) to model acute infection. CD84-deficient mice showed prolonged survival (Fig. 5d).

Taken together, these results suggest that CD84 deficiency dampens inflammation in the lung and contributes to prolonged survival after H37Rv infection.

## DISCUSSION

Concern over the length of TB treatment regimens and reductions in treatment success rates due to the increasing prevalence of antibiotic-resistant TB has renewed interest in alternative host-directed therapies (48). The success of immune checkpoint therapies that reinvigorate immune responses in cancer therapy, such as those targeting PD-1/PDL-1 (49), has prompted a search for immune cell inhibitory receptors that trigger immunosuppressive signaling pathways during M. tuberculosis pathogenesis (50) for development as similar therapeutic targets for TB. Here, we have shown that CD84, a SLAM family receptor (32), may be a suitable candidate. While the development and function of T and B lymphocytes is not affected by CD84 deficiency in the absence of infection (35), we show here that CD84 is upregulated in lung tissues from mice infected with M. tuberculosis and in PBMCs from pulmonary TB patients (Fig. 1 and 2). M. tuberculosis challenge experiments demonstrated that CD84 suppresses both T and B cells during M. tuberculosis pathogenesis: CD84-deficient mice had elevated T cell numbers, activated T cells, and release of certain TB-related cytokines, especially IFN-γ (Fig. 3), as well as increased B cell activation, IgA production, and GC B cell clustering (Fig. 4). CD84-deficient mice in an acute M. tuberculosis challenge model also showed improved M. tuberculosis clearance and longer survival than M. tuberculosis-infected WT mice (Fig. 5). These results indicate that CD84 is likely an inhibitory receptor that is expressed on immune cells specifically during M. tuberculosis pathogenesis and promotes immunosuppressive signaling pathways, suggesting its suitability as a candidate target worthy of further investigation in the development of TB-specific checkpoint immunotherapies.

CD84 (SLAM5) is a cell surface receptor that functions as a homophilic adhesion molecule and is expressed on almost all leukocyte subsets (32). CD84-mediated signaling is reported to regulate diverse immunological processes, including T cell cytokine secretion, monocyte activation, and cognate T cell-B cell interactions in the GC (32, 35, 51). Previous studies have indicated its involvement in autoimmune and lymphoproliferative disorders (47), and specific allelic variations in CD84 have been associated with autoimmune disorders, such as systemic lupus erythematosus and rheumatoid arthritis (52, 53). To our knowledge, this is the first report of the involvement of CD84 in infectious disease-associated immune processes.

The expression level of CD84 and whether it acts as a stimulatory or inhibitory receptor depend on cell type and differentiation/activation status (54, 55). Signals from costimulatory/coinhibitory receptors and their ligands determine activation or inhibition and are required for T cell activation, in addition to a signal from a T cell receptor (TCR) (56). Here, the T cell immune response of M. tuberculosis-infected mice was much stronger in CD84-deficient mice than in WT mice (Fig. 3), providing evidence that CD84 acts as an inhibitory receptor during M. tuberculosis pathogenesis.

Analysis of cytokine secretion indicated the involvement of CD84-mediated signaling in the regulation of T cell cytokine secretion during M. tuberculosis pathogenesis; cytokine levels at 80 days postinfection, particularly IFN-γ levels, were elevated in M. tuberculosis-infected CD84-deficient mice relative to their levels in the WT control (Fig. 3d). IFN-γ is essential in protection against mycobacteria and is mainly secreted by Th1 cells that activate macrophages to kill M. tuberculosis by inducing the expression of nitric oxide synthase and the production of reactive nitrogen intermediates (57, 58). CD8^+^ T cells contribute to protection also, by lysing infected alveolar macrophages (59), and they also release IFN-γ. The elevated IFN-γ levels observed here both in M. tuberculosis-infected CD84-deficient mice (at 80 days postinfection) and in CD84^−^ CD4^+^ T cells from TB patients may be related to the increased levels of Th1 or CD8^+^ T cell differentiation detected (Fig. 3).

The involvement of other coinhibitory receptors in M. tuberculosis pathogenesis has been investigated (19). For example, when CTLA-4 (CD152), an immune checkpoint previously shown to downregulate immune responses (60), is blockaded in mice, mycobacterial-infection-induced lymphocyte expansion and effector cell cytokine production in the draining lymph node are enhanced (11). Blockade of TIM-3, an immune checkpoint reported to play an important role in T cell exhaustion (9), restores T cell functions and improves control of bacterial loads in chronically M. tuberculosis-infected susceptible mice (9). These coinhibitory molecules, however, unlike CD84, also function as inhibitory receptors in the absence of infection (5). As mentioned above, CD84 is reported not to be crucial for the normal development and function of T and B lymphocytes (35). Previous investigations, however, have overlooked the need to examine the effect of infection status on CD84 expression levels. Our results suggest that CD84 has an inhibitory role during M. tuberculosis pathogenesis in mice (Fig. 3), making it an attractive target for further investigation in the development of TB-specific immune checkpoint therapies.

Effective immunity against invading pathogens depends on the continuous generation of antibodies that facilitate clearance. GCs are important sites of antibody affinity maturation and are formed during immune responses (61), the great majority of their cells being activated B cells. While CD84 deficiency does not affect bone marrow cellularity, composition, and B cell development in mice, CD84 does modulate T and B cell dynamics in GCs and is required for maximal GC formation and T cell-dependent antibody production (35). Here, we have shown that CD84 has an inhibitory role on B cell function in M. tuberculosis-infected mice; M. tuberculosis-infected CD84-deficient mice displayed higher levels of B cell activation and IgA production, possibly due to the increased area occupied by GC B cell clusters in these mice compared to that in WT mice (Fig. 4).

The development of immunotherapies that target T cell cosignaling is now a major focus within immunotherapy research (62, 63), and the successful use of PD-1 and CTLA-4-specific monoclonal antibodies in cancer treatments has provided clear evidence of its efficacy (22, 64). Currently used cancer checkpoints, however, are not appropriate targets for TB therapy; cases of patients undergoing treatment with PD-1 checkpoint inhibitors developing either acute or reactivated TB have been reported (65), and survival is greatly reduced in M. tuberculosis-infected PD-1-deficient mice (24). A suitable checkpoint target for use in TB therapy will be an inhibitory receptor that is specifically expressed at elevated levels under M. tuberculosis infection. Not only was CD84 expressed here at higher levels under M. tuberculosis infection, but CD84-deficient mice also showed stronger T and B cell immune responses during M. tuberculosis pathogenesis (Fig. 3a and b).

Our study has some limitations. Our M. tuberculosis challenge experiments were designed to establish relatively severe disease with high lung bacterial burdens within a short time frame. We chose to use C57BL/6 mice and intravenous M. tuberculosis infection in order to achieve this goal (66), rather than the more widely used aerosol infection model (67). While the aerosol infection model is likely a better model of natural infection, the intravenous model has been widely used and has the advantage of giving rise to more severe disease within a shorter time frame (68). The results obtained using human PBMCs from pulmonary TB patients (Fig. 1 to 3) were in general agreement with those from our murine experiments, providing confidence that the TB infection model established here was effective for investigating TB disease. One further limitation is that we have not yet had the opportunity to evaluate the impact of CD84 blockade (with small interfering RNA [siRNA], an inhibitor, or an antibody) on immune responses during M. tuberculosis pathogenesis. Such data would provide greater evidence of the value of CD84 blockade for therapeutic applications.

The identification of a suitable TB-specific checkpoint would be of significant benefit for the development of immunotherapies to improve host immunity and increase bacterial clearance, irrespective of the drug resistance status of infecting M. tuberculosis isolates. Our study suggests that CD84 has an immunosuppressant role during M. tuberculosis pathogenesis and is likely an M. tuberculosis-specific immune checkpoint worthy of further investigation. It will be important to identify neutralizing antibodies that block CD84 and CD84 self-interactions during M. tuberculosis pathogenesis and to examine whether CD84 blockade can reactivate immune cells during M. tuberculosis pathogenesis. Our work provides a strong foundation for the development of such a TB-specific immune checkpoint blockade immunotherapy. The rise in antibiotic-resistant TB increases the urgency of developing host-directed immunotherapies to strengthen host immune responses that will enhance bacterial clearance and, thus, improve patient outcomes.

## MATERIALS AND METHODS

### Study design.

To identify potential TB-specific inhibitory receptors as candidate TB checkpoint targets, we first performed transcriptome analysis to observe the expression levels of known cosignaling receptors in the mouse lung during M. tuberculosis pathogenesis. Having shown that the expression of SLAM family receptors was significantly upregulated, we first confirmed this finding using qPCR and flow cytometry. We then used flow cytometry to examine the expression of CD84 on immune cells during M. tuberculosis pathogenesis (in both mice and human PBMCs). To investigate the role of CD84 in T cell and B cell immune responses during M. tuberculosis pathogenesis, we performed further M. tuberculosis challenge experiments using CD84-deficient mice and also investigated the levels of IFN-γ release from CD84^−^ PBMCs from pulmonary TB patients. We investigated whether CD84 is an inhibitory receptor (i.e., its deficiency activates cell immune responses more markedly during M. tuberculosis pathogenesis) by examining T cell numbers, activation status, and cytokine secretion and B cell activation, IgA production, and GC B cell clustering in M. tuberculosis-infected CD84-deficient mice. Finally, we investigated the likely significance of the CD84 receptor in disease progression, measuring the bacterial burdens and survival in M. tuberculosis-infected CD84-deficient mice compared to those in WT mice.

### Study participants.

Whole blood was obtained from healthy donors (*n* = 20) and pulmonary TB patients (*n* = 40) at the Hunan Chest Hospital (Changsha, China). Healthy participants were adults over 18 years of age who were serologically confirmed to be M. tuberculosis negative. All TB patients had not undergone any anti-TB treatment at the time of recruitment. Detailed clinical characteristics are shown in Table S2. All study participants in this cohort were recruited at the same time. Clinical sample collection was approved by the Ethics Committee of the Hunan Chest Hospital, China.

### Animal care.

C57BL/6 mice were purchased from Cyagen Biosciences (Cyagen Biosciences, Inc., Suzhou, China). All mice were housed under specific-pathogen-free conditions in the animal care facilities at the Beijing Chest Hospital (Beijing, China). All animal procedures were approved by the Ethics Committee of Beijing Chest Hospital, Capital Medical University (Beijing, China) and were performed in accordance with the Chinese Council on Animal Care guidelines (69).

### Generation of CD84-deficient mice.

CD84-deficient mice were generated using CRISPR/Cas9 technology in a C57BL/6N mouse genetic background (Fig. S1). A site in exon 3 of this 9-exon gene was targeted by transfecting cells with a plasmid coexpressing codon-optimized Cas9 nuclease and the guide RNA targeting CD84. The genotype of offspring was verified by PCR and sequencing analysis using genomic DNA extracted from the tails of newborn pups.

### Bacterial culture, infection of mice, and CFU assays.

M. tuberculosis H37Rv (ATCC 25618) was grown in Middlebrook 7H9 broth (BD Difco Laboratories, Sparks, MD) supplemented with OADC (oleic acid, albumin, dextrose, and catalase) and cultured at 37°C in an atmosphere of 5% CO_2_. CD84-deficient and wild-type mice were intravenously infected (tail vein) with ∼1 × 10^6^ CFU M. tuberculosis H37Rv in the animal facilities at Beijing Chest Hospital. Mice were sacrificed at the time points indicated, pulmonary homogenates were prepared, and cells were washed three times with phosphate-buffered saline (PBS) to remove extracellular bacteria. After lysing cells with sterile water containing 0.01% Triton X-100, 10-fold dilutions of each lysate were cultured on 7H10 agar plates for 21 days. CFU were counted to determine bacterial survival rates.

### Transcriptome generation, functional annotation, and classification.

Total RNA from lung tissues of WT and CD84-deficient mice infected with ∼1 × 10^6^ CFU M. tuberculosis H37Rv for 30 days (three biological replicates) was prepared using TRIzol (Invitrogen, Carlsbad, CA, USA) according to the manufacturer’s instructions, resuspended in RNase-free water, and stored at −80°C. mRNA was fragmented and converted into a strand-specific RNA-Seq library using KAPA stranded RNA-Seq kits (KAPA Biosystems, USA) according to the manufacturer’s instructions. For both cDNA and genomic libraries, cluster generation was performed on an Illumina C-bot (Illumina, San Diego, CA, USA) and 2 × 150-bp paired-end sequencing was performed using an Illumina HiSeq X Ten (Illumina, San Diego, CA, USA). The quality of raw sequencing data was checked by fastQC (https://www.bioinformatics.babraham.ac.uk/projects/fastqc/). Low-quality reads were filtered using Trimmomatic 0.36 (70), and STAR (71) was used to map filtered reads to the human reference genome (GRCh38). Duplicate reads were filtered using Picard Tools 2.1.0 (72). Differential genes were determined using the DEseq package (73) in R, with cutoff parameters set at fold change: 2; *P < *0.05. Figures were plotted using ggplot2 (https://ggplot2.tidyverse.org/). Gene Ontology (GO) analysis (https://david.ncifcrf.gov/tools.jsp) was performed to obtain functional classifications, and KEGG pathway analysis (http://www.genome.jp/kegg/) was performed to determine the involvement of differentially expressed genes in different biological pathways.

### Quantitative PCR.

Total RNA was extracted from purified B cells and T cells sorted with mouse anti-CD4, anti-CD8, and anti-CD19 MicroBeads (Miltenyi Biotec, Bergisch Gladback, Germany), using TRIzol (Invitrogen, Carlsbad, CA, USA) according to the manufacturer’s instructions. cDNA was synthesized using the SuperScript III first-strand synthesis system (Invitrogen) according to the manufacturer’s instructions. Quantitative PCR was performed using SYBR select master mix (Thermo Fisher Scientific, Waltham, MA, USA) on a QuantStudio7 (Applied Biosystems, Waltham, MA, USA). All reactions were performed in a total reaction mixture volume of 10 μL, with initial denaturation at 95°C for 2 min, followed by 40 PCR cycles consisting of annealing at 55°C for 15 s and extension at 72°C for 30 s, and then final denaturation at 95°C for 30 s. Gene expression levels were normalized to the level of the glyceraldehyde-3-phosphate dehydrogenase (GAPDH) gene. The primers used are listed in Table S3.

### Preparation of purified PBMCs and *in vitro* cell stimulation assays.

PBMCs from healthy donors and TB patients were collected by Ficoll-Paque density gradient centrifugation of 5-mL amounts of heparinized blood samples. Amounts of 5 × 10^5^ cells per well were seeded into 96-well plates and plated in RPMI 1640 medium (Gibco, Paisley, UK) supplemented with 10% fetal bovine serum (Gibco, Paisley, UK). Cells were stained with phycoerythrin (PE)–anti-CD84 antibody and purified using anti-PE microbeads (Miltenyi Biotec, Bergisch Gladback, Germany). Sorted cells were incubated with either soluble anti-CD3 antibody (clone OKT3) or ESAT-6 (5 μg/mL). The concentrations of IFN-γ in the supernatants were determined using an ELISA Max deluxe set human IFN-γ kit (BioLegend, San Diego, CA, USA) according to the manufacturer’s instructions.

For the Th1 differentiation assay, naive CD4^+^ T cells were sorted using a MojoSort human CD4 naive T cell isolation kit (BioLegend, San Diego, CA, USA) and cultured at 37°C in 5% CO_2_ for 5 days in the presence of 3 μg/mL anti-CD3 antibody (clone OKT3), 3 μg/mL anti-CD28 antibody (clone 28.2), 10 μg/mL anti-IL-4 antibody (clone MP4-25D2), and 5 ng/mL IL-2. All antibodies were purchased from BioLegend (San Diego, CA, USA). Cells were stained by peridinin chlorophyll protein (PerCP)–anti-CD4 antibody (OKT4) and PE–anti-IFN-γ antibody (4S.B3) and detected by flow cytometry following stimulation with 2 μg/mL cell activation cocktail (BioLegend, San Diego, CA, USA) for 6 h.

### Detection of lymphocyte cell surface receptors by flow cytometry.

Detection of CD84 or CD69 expression on lung-/spleen-derived CD4^+^ and CD8^+^ T cells and B cells was performed as follows. In brief, lung or spleen tissue from mice sacrificed at the time points indicated was macerated through a 70-μm cell strainer using a sterile syringe plunger and washed with 10 mL PBS. Cells were centrifuged at 400 × *g* for 10 min and resuspended in 2 mL ammonium-chloride-potassium (ACK) red blood cell lysis buffer (Thermo Fisher Scientific, Waltham, MA, USA) for 5 min. Ten milliliters of PBS was added to halt the reaction, and cells were centrifuged for 10 min at 400 × *g*. The lymphocytes obtained were washed in fluorescence-activated cell sorting (FACS) buffer (PBS, 2% heat-inactivated fetal bovine serum [FBS], and 10 mM NaN_3_) and stained using fluorescein isothiocyanate (FITC)–anti-CD69 antibody (H1.2F3), PE–anti-CD84 antibody (mCD84.7), PerCP–anti-CD4 antibody (RM4-5), allophycocyanin (APC)–anti-CD8 antibody (53-6.7), and APC–anti-CD19 antibody (6D5). Human PBMCs were stained using FITC–anti-CD69 antibody (H1.2F3), PE–anti-CD84 antibody (CD84.1.21), PerCP–anti-CD4 antibody (OKT4), APC–anti-CD8 antibody (SK1), and APC–anti-CD19 antibody (HIB19). All antibodies were purchased from BioLegend (San Diego, CA, USA), and signals were detected using a BD FACSCalibur (BD Biosciences, San Jose, CA, USA). Data were analyzed using FlowJo (Tree Star, Inc., Ashland, OR, USA).

### Histopathology and immunofluorescence analysis.

Tissues were fixed in 4% paraformaldehyde and stored at 4°C. Samples for histological analysis were dehydrated and held in paraffin overnight. Sections (5 to 6 μm) were cut, mounted on glass slides, and dried overnight at 37°C. Sections were rehydrated and stained with hematoxylin and eosin Y (H&E). For acid-fast staining, lung sections were stained with carbol fuchsin and then decolorized with 3% HCl in 95% alcohol and counterstained with methylene blue. The total area occupied by inflammation was calculated to quantify the severity of lung injury. Five squares on the lung section image were selected at random to calculate the total area occupied by inflammation. The percentage of the area occupied by inflammation was calculated by dividing the total area occupied by inflammation by the total lung section area. Stained sections were analyzed with a Leica DM4000 fluorescence microscope and Leica software (Leica Microsystems, Buffalo Grove, IL).

Lung and spleen cryostat sections (∼5 to 6 μm) were prepared (as described above) for GC analysis and stained using the following antibodies and reagents: PE–anti-IgD and PerCP–anti-IgD (11-26c.2a) and FITC–anti-GL7 antibody (GL7). All antibodies were purchased from BioLegend (San Diego, CA, USA). The numbers of GCs (GL-7^+^ IgD^−^ areas in the follicular mantle) were determined by systematic examination of each lung/spleen section. GC images were captured on an LSM-700 confocal microscope (Zeiss, Jena, Germany) at ×20 magnification using Zeiss AIM (version 4.2) software (Zeiss, Jena, Germany) to determine the GC area.

### Cytokine analysis.

The levels of selected cytokines and chemokines in serum from sacrificed mice were determined with a multiplexed sandwich ELISA quantitative array (Quantibody mouse cytokine array 1 kit; Raybiotech, Inc., Guangzhou, China) according to the manufacturer’s instructions. Signals were visualized using an InnoScan 300 microarray scanner (Innopsys, Carbonne, France). Serum levels of IFN-γ were further validated using a RayBio mouse IFN-γ kit (Raybiotech, Inc., Guangzhou, China) according to the manufacturer’s instructions, the results being read on a Biotek Elx800 microplate reader (Biotek, Winooski, VT, USA).

### Statistical analysis.

Statistical analysis was performed using GraphPad Prism software (GraphPad Software, Inc., San Diego, CA, USA). Data are expressed as the mean values ± standard deviations (SD). All *P* values were calculated using two-tailed unpaired *t* tests. A *P* value of <0.05 was considered statistically significant (*, *P* < 0.05; **, *P* < 0.01; and ***, *P* < 0.001).

### Data availability.

The raw sequence data reported in this paper have been deposited in the Genome Sequence Archive (74), the National Genomics Data Center (75), and the China National Center for Bioinformation/Beijing Institute of Genomics, Chinese Academy of Sciences, under accession number CRA004502 and are publicly accessible at https://ngdc.cncb.ac.cn/gsa.
