# Supplementary material for: CD84 is a Suppressor of T and B Cell Activation during Mycobacterium tuberculosis Pathogenesis
Source: Microbiol Spectr. 2022 Feb 23;10(1):e01557-21. doi: 10.1128/spectrum.01557-21 (PMC8865571; doi:10.1128/spectrum.01557-21)
Supplement: SUPPLEMENTAL FILE 1 — Supplemental material. Download SPECTRUM01557-21_Supp_1_seq1.pdf, PDF file, 0.6 MB [file spectrum01557-21_supp_1_seq1.pdf]

Supplementary materials

for

CD84 is a suppressor of T and B cell activation during *Mycobacterium tuberculosis* pathogenesis

Nan Zheng[1,5], Joy Fleming[1], Peilei Hu[2], Jianjian Jiao[1], Guoqin Zhang[1], Ruifang Yang[3], Chuanyou Li[3], Yi Liu[3], Lijun Bi[1,4]*, Hongtai Zhang[1]*

[1] Key Laboratory of RNA Biology and State Key Laboratory of Biomacromolecules, CAS Center of Excellence in Biomacromolecules, Institute of Biophysics, Chinese Academy of Sciences; Beijing 100101, China
[2]Hunan Chest Hospital, Changsha 410013, Hunan Province, China
[3]Beijing Chest Hospital, Capital Medical University, Beijing Tuberculosis and Thoracic Tumor Research Institute, Beijing Key Laboratory for Drug Resistant Tuberculosis Research; Beijing 101149, China.
[4]Guangdong Province Key Laboratory of TB Systems Biology and Translational Medicine, Foshan 528000, Guangdong Province, China.
[5]University of Chinese Academy of Sciences; Beijing 100049, China

Supplementary Tables

Table S1. Changes in gene expression levels of T cell co-stimulatory and co-inhibitory receptors in the transcriptome of MTB-infected (1×10^6 CFUs H37Rv) C57BL/6 mice compared to uninfected controls, 30 d post-infection

Table S2. Demographic characteristics of the study participants

Table S3. Primer sequences used in this study

Supplementary Figures

Fig. S1. Generation of CD84-deficient C57BL/6N mice

Fig. S2. CD84 deficiency does not affect T cell numbers or activation state

Fig. S3. CD84 deficiency does not affect B cell numbers

Fig. S4. Immunohistology of lung tissue from MTB-infected (1×10^6 CFUs H37Rv) WT and CD84-deficient C57BL/6 mice, 60 d post-infection.

Table S1. Changes in gene expression levels of T cell co-stimulatory and co-inhibitory receptors in the transcriptome of MTB-infected ($1{\times}10^6$ CFUs H37Rv) C57BL/6 mice compared to uninfected controls, 30 d post-infection

Receptor subfamily	Receptor subfamily	Upregulated / downregulated / no change
CD28	CD28	upregulated
	ICOS (CD278)	upregulated
	CTLA4 (CD152)	upregulated
	PD1 (CD279)	upregulated
	PD1H (VISTA)	no change
	BTLA (CD272)	upregulated
B7	B71 (CD80)	upregulated
	B7H1 (CD274, PDL1)	upregulated
CD226	CD226 (DNAM1)	upregulated
	CRTAM (CD355)	upregulated
	TIGIT (VSIG9, VSTM3)	upregulated
	CD96 (TACTILE)	upregulated
TIM	TIM1 (HAVCR1, KIM1)	no change
	TIM2 (TIMD2)	no change
	TIM3 (HAVCR2, KIM3)	upregulated
	TIM4 (TIMD4)	upregulated
CD2/ SLAM	CD2 (LFA2, OX34)	upregulated
	SLAM (CD150, SLAMF1)	upregulated
	2B4 (CD244, SLAMF4)	upregulated
	Ly108 (NTBA, CD352, SLAMF6)	upregulated
	CD84 (SLAMF5)	upregulated
	Ly9 (CD229,SLAMF3)	upregulated
	CRACC (CD319, BLAME, SLAMF7)	upregulated
BTN	BTN1 (BTN1A1)	no change
	BTN2 (BTN2A1-3)	no change
	BTN3 (BTN3A1-3)	no change
LAIR	LAIR1	upregulated
Orphan	Orphan LAG3 (CD223)	upregulated
	CD160 (BY55, NK28)	upregulated

Table S2. Demographic characteristics of the study participants

	**Active TB**	**Control**
Number of participants	40	20
Age (years), mean ± SD	46.6 ± 17.54	41.1 ± 18.21
Age range (years)	15-86	14-81
Gender (male/female)	15/25	5/15

Table S3. Primer sequences used in this study

Primer name	Sequence	Notes
Forward primer (F1)	5’-GGGTTGATGAAGTATTGGGACAC-3’	knock-out screening
Reverse primer (R1)	5’-GAATCAGGAGAGACAATGAAGAAAG-3’	
Forward primer (F2)	5’-AGGTCTAACTTTACTTCTGGCCTTG-3’	
Internal control PCR primer F	5’-CTATCAGGGATACTCCTCTTTGCC-3’	
Internal control PCR primer R	5’-GATACAGGAATGACAAGCTCATGGT-3’	
GAPDH forward primer	5’-AGGTCGGTGTGAACGGATTTG-3’	RT–qPCR
GAPDH reverse primer	5’-GGGGTCGTTGATGGCAACA-3’	
SLAMF1 forward primer	5’-GTCAGCAGTATCTCTAGGACC-3’	
SLAMF1 reverse primer	5’-CATCCATGGACTCGATTCTG-3’	
Ly9 forward primer	5’-CAAAGCCTGGAATCCAGTC-3’	
Ly9 reverse primer	5’-TTTCTTCTGGAGGCTCCTG-3’	
CD84 forward primer	5’-TACAAACCTGGTCTGAAGC-3’	
CD84 reverse primer	5’-GAGGAAAGTAACTGACTCCC-3’	

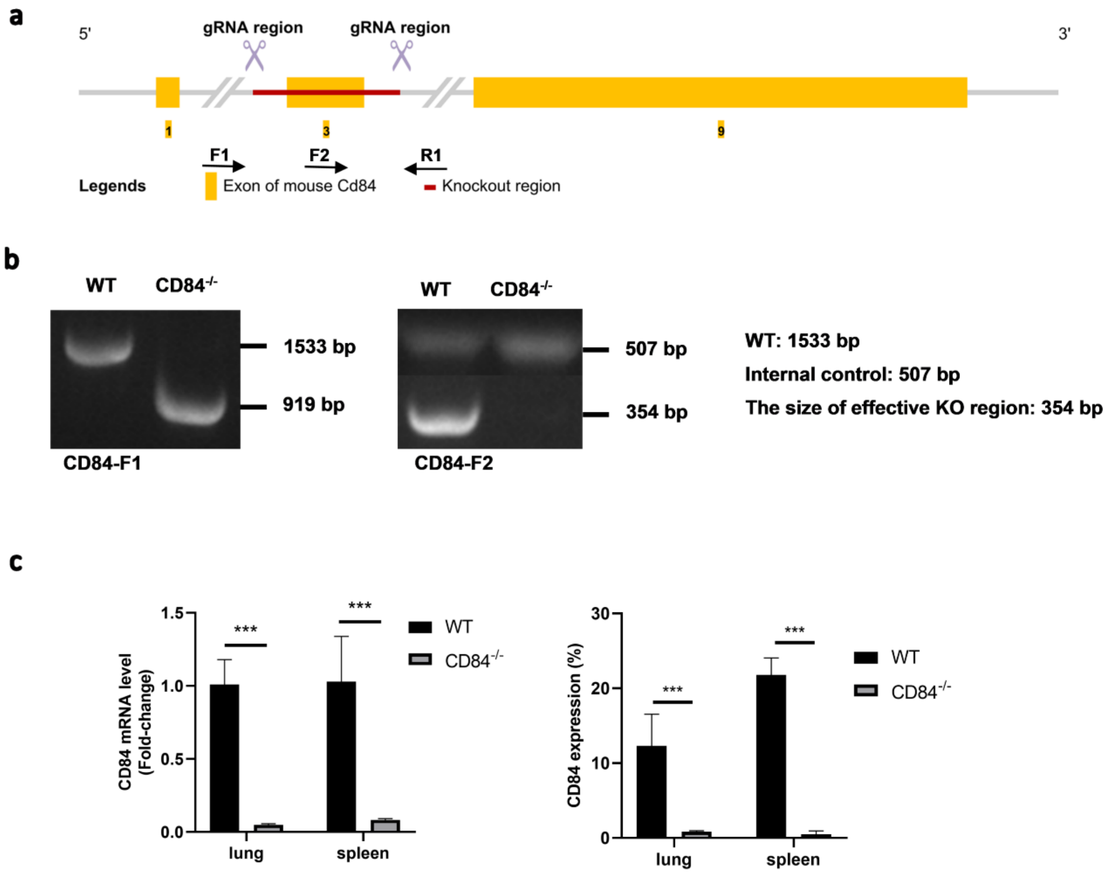

Fig. S1. Construction of CD84-deficient C57BL/6N mice. (a) The CD84 gene (NCBI Reference Sequence: NM_013489; Ensembl: ENSMUSG00000038147) located on mouse chromosome 1 has 9 exons. Exon 3 was selected as the target site for editing. Cas9 and gRNA were co-injected into fertilized eggs for CD84-deficient mouse production. **(b)** Genotyping of CD84-deficient pups was performed by PCR using genomic DNA extracted from pup tails. The primers are listed in Table S3. **(c)** qPCR determination of CD84 mRNA levels in WT and CD84-deficient mice (left) and flow cytometry determination of the percentage of $CD84^+$ cells in lung and spleen tissues from WT and CD84-deficient mice.

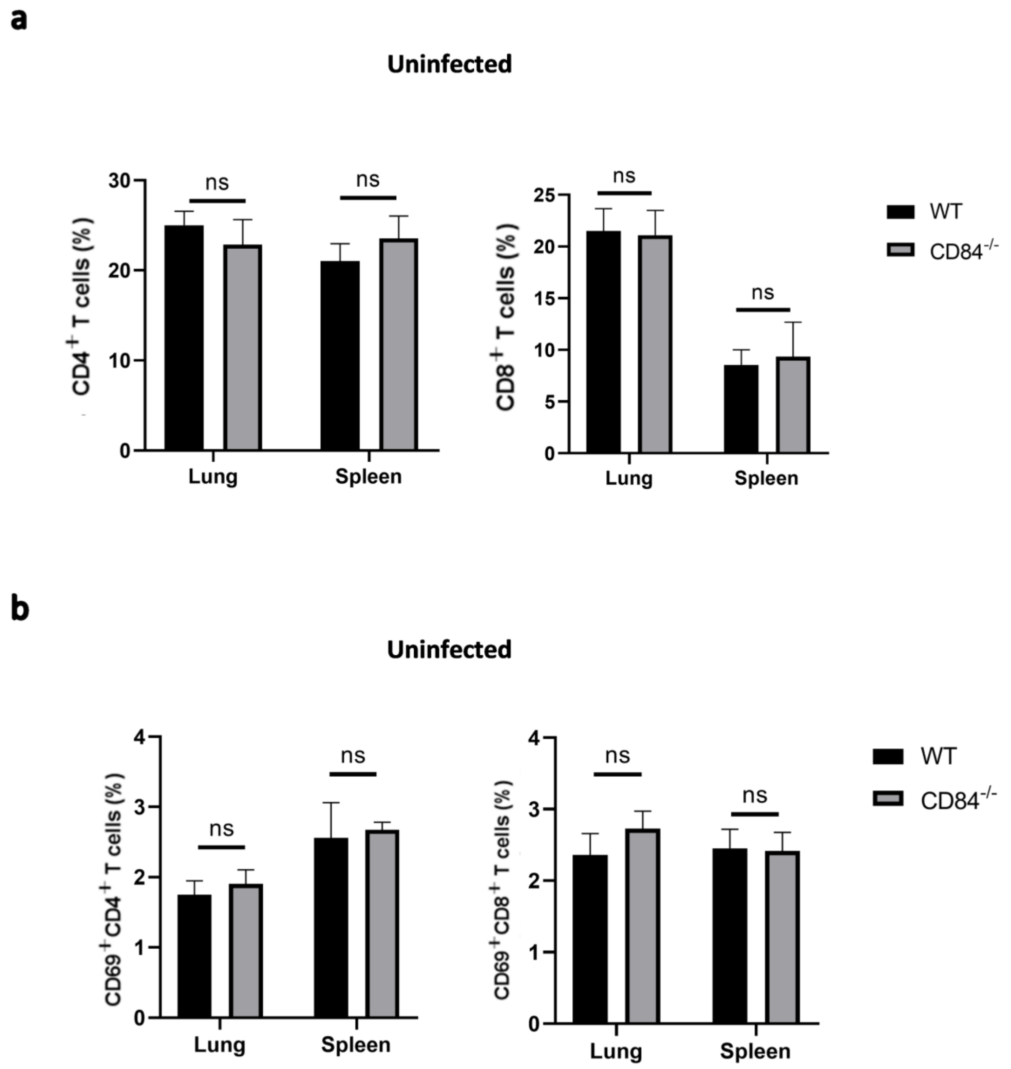

Fig. S2. CD84 deficiency does not affect T cell numbers or activation state. T cells were separated from the lung and spleen tissues of uninfected WT and CD84-deficient C57BL/6 mice. **(a)** Percentage of $CD4^+$ and $CD8^+$ T cells in lung and spleen tissues from uninfected WT and CD84-deficient mice, as determined by flow cytometry. **(b)** Percentage of CD69-expressing $CD4^+$ and $CD8^+$ T cells from uninfected WT and CD84-deficient mice, as determined by flow cytometry. Data correspond to the mean ± SD from three independent experiments, n = 3 for each group in each experiment. ns = non-significant, Student's *t*-test.

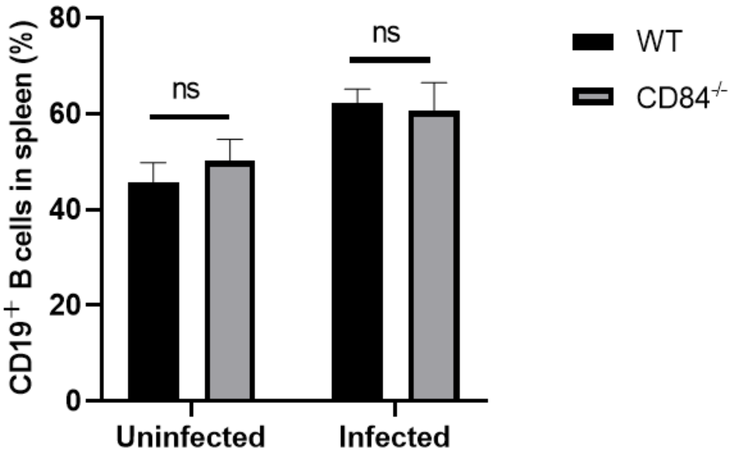

Fig. S3. CD84 deficiency does not affect B cell numbers. B cells were separated from spleen tissues from MTB-infected (1×10^6 CFUs H37Rv) and uninfected WT and CD84-deficient C57BL/6 mice, 60 d post-infection. The percentage of B cells (among all lymphocytes) was determined by flow cytometry. Data correspond to the mean ± SD from three independent experiments, n = 3 for each group in each experiment. ns = non-significant, Student's *t*-test.

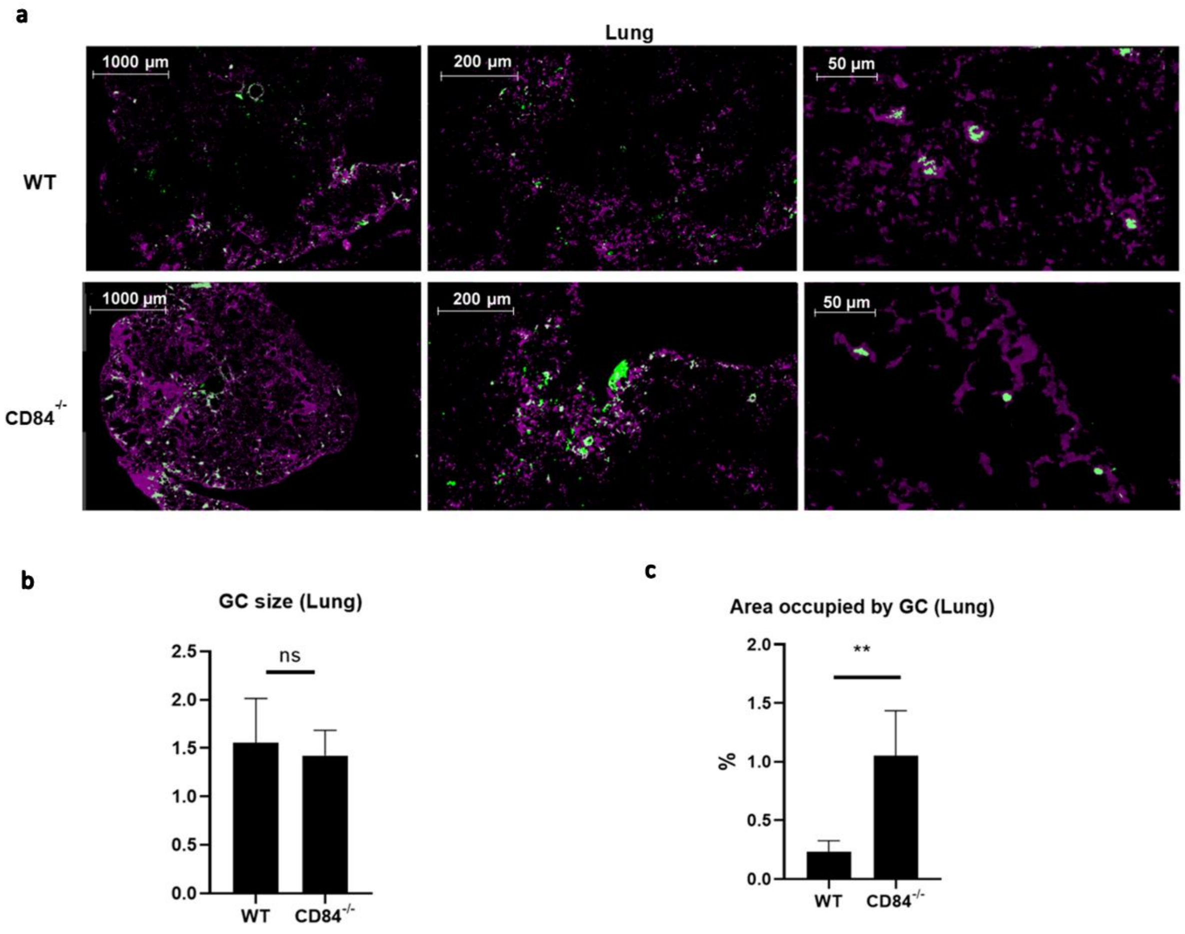

Fig. S4. Immunohistology of lung tissue from MTB-infected (1×10^6 CFUs H37Rv) WT and CD84-deficient C57BL/6 mice, 60 d post-infection. (a) Immunohistology of lung tissue stained with GC B cell markers Percp-IgD (purple) and FITC-GL7 (green). **(b)** Average GC size and **(c)** Percentage area occupied by GC as quantified using a Zeiss Axioplan microscope morphometric tool. Data presented are means ± SD from three independent experiments, n = 3 for each group in each experiment. **: p <0.01, ns: non-significant, Student's *t*-test.
